# Profile of health professionals who completed a master's, doctoral, or post-doctoral degree in one Brazilian pediatric program

**DOI:** 10.6061/clinics/2020/e1392

**Published:** 2020-04-13

**Authors:** Clovis Artur Silva, Vitor Cavalcanti Trindade, Amanda Monteiro da Cruz, Bruna Paccola Blanco, João Fernando Vecchi Santos, Alexandre Archanjo Ferraro, Vicente Odone-Filho, Uenis Tannuri, Werther Brunow Carvalho, Magda Carneiro-Sampaio, Sandra Elisabete Vieira, Sandra Josefina Ferraz Ellero Grisi

**Affiliations:** I Instituto da Crianca e do Adolescente (ICr), Hospital das Clinicas HCFMUSP, Faculdade de Medicina, Universidade de Sao Paulo, Sao Paulo, SP, BR

**Keywords:** Master's Degree, Doctoral Degree, Post-Doctoral Degree, Salary, Faculty

## Abstract

**OBJECTIVE:**

This study aimed to determine the personal and professional characteristics, and the physical, psychiatric/psychological, and professional issues that exist among master's-, doctoral-, and post-doctoral-level health professionals.

**METHODS:**

A cross-sectional, online, self-reported survey of 452 postgraduates who completed master's, doctoral, or post-doctoral degrees in one graduate program in pediatrics in São Paulo, Brazil, was conducted.

**RESULTS:**

The response rate was 47% (211/453). The majority of participants were women (78%) and physicians (74%), and the median age was 47 years (28-71). Master's, doctoral, and post-doctoral degrees were reported by 73%, 53%, and 3%, respectively. High workload (>40 hours/week) occurred in 59%, and 45% earned ≥15 minimum wages/month. At least one participation in scientific meeting in the past year was reported by 91%, and 79% had published their research. Thirty-nine percent served as a member of a faculty of an institution of higher learning. The data were analyzed by two age groups: participants aged ≤48 years (group 1) and participants aged >48 years (group 2). The median rating of overall satisfaction with the profession in the past year [8 (0-10) *vs*. 9 (1-10), *p*=0.0113]; workload >40 hours/week (53% *vs*. 68%, *p*=0.034); and ≥15 minimum wages/month (37% *vs*. 56%, *p*=0.0083) were significantly lower in group 1. Further analysis by gender revealed that the median rating of overall satisfaction with the profession in the past year [8 (0-10) *vs*. 9 (3-10), *p*=0.0015], workload >40 hours/week (53% *vs*. 83%, *p*=0.0002), and ≥15 minimum wages/month (37% *vs*. 74%, *p*=0.0001) were significantly lower in women compared with men. The median rating of overall satisfaction with the mentorship supervision provided was significantly higher among the women 10 (5-10) *vs*. 10 (2-10), *p*=0.0324].

**CONCLUSIONS:**

The majority of master's-, doctoral-, and post-doctoral-level health professionals were women and physicians, and had published their thesis. Younger postgraduates and women reported low salaries, less likelihood of working >40 hours/week, and less overall satisfaction with their profession. Further longitudinal and qualitative studies are warranted to assess career trajectories after graduation.

## INTRODUCTION

Contemporary teaching methods, humanistic approaches to patient care, and early exposure to scientific research are common in undergraduate, fellowship, residency, and postgraduate programs 1-9. The career development of health professionals after completion of undergraduate and residency training varies around the world 9-14.

In Brazil, the University of São Paulo is a major producer of highly qualified members of the workforce for teaching, research, and science and technology. The university is responsible for educating a significant portion of the country's master's, doctoral, and post-doctoral students 15. The Pediatric Department at the university has a master's/doctoral and post-doctoral program, which is responsible for epidemiological, translational, and clinical research in children and adolescent health, focusing on primary, secondary, and tertiary care; subspecialties; and transplantation. The program includes professors and students and involves physicians and non-physicians (e.g., physiotherapists, dietitians, nurses, physical educators, biomedicals, and dentists).

Previous research has shown that personal, professional, and educational characteristics, ratings of overall satisfaction, physical/psychological stress, and professional issues after undergraduate, fellowship, and residency training are different around the world 5,6,16-18. However, to the best of our knowledge, simultaneous analysis of these data has not been carried out among health professionals in Latin America, after master's, doctoral, and post-doctoral degree completion.

Thus, the objective of the present study was to evaluate postgraduate reports regarding demographic characteristics, overall satisfaction, salary, teaching, publishing, patient care profiles, and main physical, psychiatric/psychological and professional issues related to clinical practice. Additionally, personal, professional, and educational characteristics and issues were compared by age and gender.

## METHODS

A cross-sectional, online, self-reported survey of 452 individuals who had successfully completed a master's, doctoral, or post-doctoral degree in a pediatric postgraduate program at a university in São Paulo, Brazil, was conducted to collect data on demographic, professional, and educational characteristics and physical, psychiatric/psychological, and professional issues among postgraduate health professionals. In this postgraduate program, 3 years are required to complete the master's degree, and up to 4 years are required to complete the doctoral degree. The program focuses on epidemiological, translational, and clinical research in pediatric primary, secondary, and tertiary care; subspecialties; and transplantation.

The survey was carried out using Research Electronic Data Capture (REDCap; Vanderbilt University, Nashville, TN, USA), which is a safe Web application for developing research databases that can also be used for building, managing, and accessing electronic surveys. Between January and February 2019, all health professionals that had completed their master's, doctoral, or post-doctoral degree in the pediatric postgraduate program between 1995 and 2018 were invited to participate in the survey. The ethics committee of the university hospital approved this study (CAAE: 93564518.5.0000.0068).

Invitations to participate in the anonymous, self-reported survey were distributed by email. The survey included 22 questions on demographic, professional, and educational characteristics, and physical, psychiatric/psychological, and professional issues. The formats of the questions were multiple choice, dichotomous (yes/no), or horizontal visual analog scale. The questions referred to events during and after degree completion. Estimated time to complete the questionnaire was approximately 15 minutes.

The topics of the 22 items were as follows:

1. Demographic and educational data (current age; gender; ethnicity; marital status; nationality; number of children; city; state; country; year of undergraduate completion; major course; years since completion of master's, doctoral, or post-doctoral degree; master's, doctoral, or post-doctoral mentorship; and profession and specialty after undergraduate completion).
2. Master's, doctoral, or post-doctoral degrees.
3. Overall satisfaction with the written research project and its submission to the Ethics Committee, measured with a horizontal visual analog scale (0=“no satisfaction at all” and 10=“complete overall satisfaction”).
4. Overall satisfaction with the process of writing the scientific manuscript, measured with a horizontal visual analog scale (0=“no satisfaction at all” and 10=“complete overall satisfaction”).
5. Overall satisfaction with the process of submitting the manuscript to a scientific journal, measured with a horizontal visual analog scale (0=“no satisfaction at all” and 10=“complete overall satisfaction”).
6. Overall satisfaction with the mentorship supervision provided, measured with a horizontal visual analog scale (0=“no satisfaction at all” and 10=“complete overall satisfaction”).
7. Overall satisfaction with your profession in the past year, measured with a horizontal visual analog scale (0=“no satisfaction at all” and 10=“complete overall satisfaction”).
8. Overall satisfaction with the postgraduate program, measured with a horizontal visual analog scale (0=“no satisfaction at all” and 10=“complete overall satisfaction”).
9. Professional practice setting in the past year (public service, private service, pediatric primary care, pediatric infirmary, pediatric emergency room, pediatric intensive care, neonate care, pediatrician private practice, public or private university, pharmaceutical industry, medical procedure, radiology service, laboratory exam, non-governmental organization, community service, elementary school/high school, and administrative service).
10. Workload in hours/week in the past year (≤20 hours, 20-40 hours, 40-60 hours, and >60 hours).
11. Number of pediatric patients weekly (≤50 patients, 50-100 patients, 100-200 patients, >200 patients, and no patient supervision).
12. Patient health insurance issues availability among your patients in the past year (public health insurance, private insurance, and/or other).
13. Salary in the past year (<15 minimum wages/month and ≥15 minimum wages/month).
14. Ages of patients seen in the past year (neonate, <10 years, 10-20 years, 20-60 years, >60 years).
15. Patient care profiles for the past year (emergency room, infirmary, pediatric intensive care, primary care, newborn, growth and development monitoring, chronic disease management, psychiatric/psychological management, contraception and gynecological counseling, licit and illicit drug use, sexually transmitted infections and pregnancy counseling, physical/psychological violence assessment, and no patient supervision).
16. Number of scientific meeting participations in the past year.
17. Scholarship during the master's, doctoral, or post-doctoral degree program (yes/no).
18. Published papers during or after the master's, doctoral, or post-doctoral degree program (yes/no).
19. Translational, epidemiological, or clinical research contribution after the master's, doctoral or post-doctoral program (yes/no).
20. Faculty service (yes/no).
21. Current participation in a master's, doctoral, or post-doctoral degree program as a mentor (yes/no).
22. Self-reported physical and professional issues related to clinical practice in the past year (long working hours, poor social life, sedentarism, overall decrease in health-related quality of life, disruption in family life, harassment, obsessive-compulsive symptoms, low salary, legal issues, workplace violence, stress induced by chief/professor, and stress induced by patient health insurance issues).

The respondents were divided into two age groups according to the mean of current age (aged ≤48 years [group 1] and aged >48 years [group 2]) and by gender.

### Statistical analysis

The Statistical Package for the Social Sciences version 13.0 (SPSS Inc, Chicago, IL, USA) was used in the analysis of the data. Results for the continuous variables were reported as median (minimum-maximum values) or mean±standard deviation, and compared by Mann-Whitney test and Student's t-test, respectively. The results for categorical variables were reported as frequency (percentage) and compared by Fisher’s exact test or Pearson chi-square test, as appropriate. Pearson or Spearman rank correlation coefficients were employed for correlations between overall satisfaction, current age, and year of degree completion. A *p*-value less than 0.05 was considered statistically significant.

## RESULTS

The survey response rate was 53% (239/453). Incomplete data were observed among 6% (27/453) of potential respondents and 0.2% (1/453) declined participation in the study. Therefore, 47% (211/453) of potential respondents completed the questionnaire and were evaluated.

The proportion of women among those who completed the questionnaire (n=211) was equal to that among those who did not participate (n=214) ([164/211 (78%) *vs*. 167/214 (78%), *p*=1.000].

Table 1 shows the data on the demographic characteristics, region of Brazil, postgraduate degree, current profession, overall satisfaction rate, location of practice, workload in hours/week, number of patients/week, salary, and patient health insurance issues reported by 211 master's-, doctoral-, and post-doctoral health professionals. The majority of the postgraduates were women (78%), and the mean age was 47.61±10.4 years. Eighty-two percent lived in Southeastern Brazil, and 74% worked as physicians. Master's degrees were reported by 73%, doctoral degrees by 53%, and post-doctoral degrees by 3%. The majority of postgraduates (65%) had worked as public service professionals within the past year, followed by those who worked as private practice pediatricians (50%), and those who worked as public/private university professors (20%). Approximately one half of postgraduates earned ≥15 minimum wages/month (Table 1).

Participation in at least one scientific meeting was reported by almost 91% of postgraduates. Seventy-nine percent reported having published research papers in health science, and 39% were faculty members at an institution of higher learning. The most prominent physical, psychiatric/psychological, and professional issues (>40%) were physical inactivity and long working hours (Table 2).

The data on the participants were also analyzed by age. Two age groups were used in the analysis: participants aged ≤48 years (group 1, n=120) and participants aged >48 years (group 2, n=91). The median age [40 (28-48) *vs*. 58 (49-71) years, *p*<0.0001], years since master's, doctoral, or post-doctoral degree [5 (1-22) *vs*. 15 (1-45) years, *p*<0.0001], and number of children [1 (0-3) *vs*. 2 (0-4), p=0.0006] were significantly lower in group 1 compared with group 2. The median rating of overall satisfaction with one's profession in the past year [8 (0-10) *vs*. 9 (1-10), *p*=0.0113] was also significantly lower in group 1 compared with group 2. Significantly fewer participants in group 1 worked for >40 hours/week (53% *vs*. 68%, *p*=0.034) and earned ≥15 minimum wages/month (37% *vs*. 56%, *p*=0.0083) compared with those in group 2 (Table 3).

An analysis of the data by gender revealed that the median rating of overall satisfaction with the mentorship supervision provided was significantly higher among women compared with men [10 (5-10) *vs*. 10 (2-10), *p*=0.0324]. In contrast, the median rating of overall satisfaction with one's profession in the past year was significantly lower in women compared with men [8 (0-10) *vs*. 9 (3-10), *p*=0.0015], and women were significantly less likely to have a workload >40 hours/week (53% *vs*. 83%, *p*=0.0002), earn ≥15 minimum wages/month (37% *vs*. 74%, *p*=0.0001), and work long hours (38% *vs*. 55%, *p*=0.045), and were significantly more likely to be paid a low salary (35% *vs*. 19%, *p*=0.0496) (Table 4).

Spearman rank correlations revealed that current age was positively correlated with overall satisfaction with one's profession in the past year (r=0.168, *p*=0.015), and years since completion of master's, doctoral, or post-doctoral degree also was positively correlated with overall satisfaction with one's profession in the past year (r=0.160, *p*=0.019). No other correlation was observed among current age, years since completion of master's, doctoral, or post-doctoral degree, and overall satisfaction rating (written research project/scientific manuscript, submitting the paper, mentorship supervision, profession in the past year, postgraduate program, *p*>0.05).

## DISCUSSION

To our knowledge, this was the first study to explore the profile of postgraduate professionals from a Latin American postgraduate program in children and adolescent health.

The strength of the present study was the simultaneous analysis of personal, professional, and educational characteristics, and physical, psychiatric/psychological, and professional issues. The inclusion of measurements of six overall satisfaction rates, linked to the postgraduate program, scientific research and publishing, and current satisfaction with one's profession also allowed a better understanding of the quality of the program and the current impact of the program on the professional lives of its graduates. The self-report and anonymous survey allowed confidentiality of data.

The main goal of our Pediatric Postgraduate Program is career development of the faculty and researchers in the area of child and adolescent health, accompanied by great innovation. This program focuses mainly on studies dedicated to epidemiologic and patient-oriented research from the fetal period to adolescence, involving socio-economic, cultural, and biological problems. In our university, master's and/or doctoral degrees are usually pursued after an undergraduate degree has been obtained for a non-physician career and after completion of a residency for physician careers.

The majority of our postgraduates live in Southeastern, Brazil, which is the richest region of the country; this region generates approximately 60% of the Brazilian gross domestic product. Forty percent of the postgraduates in the present study serve on a faculty at an institution of higher education and have careers that are research- and teaching-oriented with a true academic trajectory. However, the majority of postgraduates work as health professionals in both private and public services (dual practice), which is common in Brazil 19, and do not focus their careers on academia. The results are different in other countries in terms of the physician-scientists model. The mentor program includes both basic sciences and clinical integration research, thus promoting student development as physician-scientists who are engaged in translational research 13,20.

In the present study, physician was the profession most reported, and the vast majority of professionals were women, in agreement with past research that has observed increased numbers of women in medical schools and residency programs around the world 21,22. The result of the present study was different from that of research on MD/PhD programs in Canada and the United States, which found that men were the predominant gender 9,10,13,20.

Analysis of the data from the postgraduate program evaluation measurements, including the five ratings of overall satisfaction, indicated that the overall rating was good, because the median rating was above 8. Almost 80% of postgraduates reported that their thesis was published, reinforcing the efficiency of our program. Overall satisfaction with one's profession in the past year was low in young health professionals after degree completion. This low enthusiasm is possibly related to high workload and low salaries. In contrast, MD-PhD students of one medical school reported optimism for their future, with up 80% of the respondents describing excellent job opportunities for physician-scientists 10.

The overall satisfaction with one's profession in the past year was also low in women, who reported low salaries and workload. Interestingly, overall satisfaction with mentorship supervision was high among women, probably due to engagement, proactiveness, commitment, and a fairly good relationship with their mentor.

It is worth noting that continuing education after degree completion was found to be an important factor. The majority of participants reported attending professional conferences regularly. In fact, this population has a notable interest in scientific knowledge 1.

There were problems related to clinical practice among the postgraduates, involving sedentarism, long work hours, and poor social life that may decrease health-related quality of life. Additionally, these health professionals may be at risk for anxiety, depression, and burnout. Future studies that clarify these issues are warranted 16-18,23. Furthermore, improvements in health professionals' training and preventive programs, targeted at enhancing overall satisfaction, and psychological/psychiatric issues diagnosis should be implemented.

This study had several limitations. One limitation was the moderate response rate (47%) to the online, self-reported survey. The long length of the questionnaire, and electronic address problems might have been reasons for dropout. The cross-sectional study design and the absence of validated tools to evaluate physiological/psychiatric issues were also limitations.

In conclusion, the quality of the postgraduate program was found to be reasonable. The program's postgraduate professionals were primarily women and physicians, and had published their thesis. Young postgraduates and women reported low salaries, low workloads, and less overall satisfaction with their profession. Further longitudinal and qualitative studies are needed to assess career trajectories after graduate degree completion.

## AUTHOR CONTRIBUTIONS

Silva CA, Trindade VC, Cruz AM, Blanco BP, Santos JFV and Grisi SJFE were responsible for the study design. Silva CA, Trindade VC, Cruz AM, Blanco BP, Santos JFV, Ferraro AA, Odone-Filho V, Tannuri U, Carvalho WB, Carneiro-Sampaio M, Vieira SE, Grisi SJFE were responsible for the acquisition of data. Silva CA, Trindade VC, Cruz AM, Blanco BP, Santos JFV, Ferraro AA, Odone-Filho V, Tannuri U, Carvalho WB, Carneiro-Sampaio M, Vieira SE, Grisi SJFE were responsible for the data analysis and interpretation. Silva CA, Trindade VC, Cruz AM, Blanco BP, Santos JFV, Ferraro AA, Odone-Filho V, Tannuri U, Carvalho WB, Carneiro-Sampaio M, Vieira SE, SJFE were responsible for the manuscript preparation.

## Figures and Tables

**Table 1:** Demographic data, region of Brazil, postgraduate degree, current profession, ratings of satisfaction, professional practice setting, workload in hours/week, number of patients/week, salary and patient health insurance issues reported by master’s-, doctoral-, or post-doctoral-level health professionals.

Variable	Master’s/Doctoral/Post-doctoral degree (n=211)
Demographic data	
Current age, years	47.61±10.4
Women	164 (78)
Caucasians	176 (83)
Year after Master/PhD/post-doctoral degree conclusion	8 (1-45)
Current married/partnered	153 (72)
Number of children	2 (0-4)
Current region of Brazil	
North	5 (2)
Northeast	10 (5)
Midwest	8 (4)
Southeast	172 (82)
South	7 (3)
Out of the country	9 (4)
Postgraduate degree	
Master	155 (73)
PhD	111 (53)
Post-doctoral	6 (3)
Current profession	
Physician	156 (74)
Other allied health professionals	55 (26)
Ratings of satisfaction	
Written research project and submission to IRB	9 (0-10)
Writing the scientific manuscript	9 (0-10)
Submitting the manuscript to a scientific journal	8 (0-10)
Mentorship supervision	10 (2-10)
Your profession in the past year	9 (0-10)
The graduate program	9 (1-10)
Professional practice setting in the past year	
Public service	138 (65)
Private service	106 (50)
Pediatric primary care	9 (4)
Pediatric infirmary	22 (10)
Pediatric emergency room	23 (11)
Pediatric intensive care	16 (7)
Neonate care	10 (5)
Pediatrician private practice	108 (51)
Public university professor	42 (20)
Private university professor	44 (21)
Pharmaceutical industry	9 (4)
Medical procedure	3 (1)
Radiology service	4 (2)
Laboratory	4 (2)
Non-governmental organization	5 (2)
Elementary school/high school	2 (1)
Administrative service	14 (7)
Workload in hours/week	
<20 hours	12 (6)
20-40 hours	73 (35)
>40 hours	126 (59)
Number of patients/week	
<50 patients	96 (45)
50-100 patients	69 (33)
100-200 patients	10 (5)
>200 patients	3 (1)
No patient supervision	33 (16)
Patient health insurance issues availability	
Public	144 (68)
Private	146 (69)
Salary in the past year	
≥15 minimum wages/month	96 (45)

Results are presented as n (%) and median (range) or mean±standard deviation, IRB-institutional review board.

**Table 2:** Age groups of patients, patient care profiles, scholarship during graduate school, number of scientific meeting participations, published research papers, translational/epidemiological/clinical research, faculty service, and main issues related to clinical practice reported by master’s-, doctoral-, or post-doctoral-level health professionals.

Variable	Master’s/Doctoral/Post-doctoral degree (n=211)
Age groups of patients	
Newborn	116 (55)
28 days to <10 years	152 (72)
10-20 years	137 (65)
>20 years	39 (18)
Patient care profiles	
Emergency room	45 (21)
Infirmary	49 (23)
Pediatric intensive care	43 (20)
Newborn	55 (26)
Growth and development monitoring	99 (47)
Chronic disease management	101 (48)
Psychiatric/psychological management	15 (7)
Contraception and gynecological counseling	4 (2)
Licit and illicit drug use	5 (2)
STI and pregnancy counseling	8 (4)
Physical/psychological violence assessment	8 (4)
Scholarship during graduate school	75 (35)
Number of scientific meeting participations	
1-2	86 (41)
3-5	84 (40)
>6	21 (10)
None	20 (9)
Published thesis	166 (79)
Translational/epidemiological/clinical research	132 (62)
Faculty staff	82 (39)
Current participation as a mentor	38 (18)
Main issues related to clinical practice	
Long working hours	89 (42)
Poor social life	74 (35)
Physical inactivity	95 (45)
Overall decrease of HRQL	60 (28)
Disruption in family life	66 (31)
Harassment	9 (4)
Low income	67 (32)
Legal problem	3 (1)
Workplace violence	1 (0.4)
Stress related to relationship with chief/professor	38 (18)
Stress related to patient health insurance issues	11 (5)

Results are presented as n (%), HRQL-health-related quality of life, STI-sexually transmitted infection.

**Table 3:** Demographic data, ratings of satisfaction, workload, patients/week, salary, scientific meeting participation, published research paper, faculty service and main issues related to profession according to years of clinical practice in two groups of master’s-, doctoral-, or post-doctoral-level health professionals: group 1 (≤48 year) and group 2 (>48 year).

Variable	Group 1 (n=120)	Group 2 (n=91)	*p*
Demographic data			
Current age, years	40 (28-48)	58 (49-71)	**<0.0001**
Women	98 (82)	66 (72)	0.134
Years since master’s/doctoral/post-doctoral completion	5 (1-22)	15 (1-45)	**<0.0001**
Currently married/partnered	85 (71)	68 (75)	0.641
Number of children	1 (0-3)	2 (0-4)	**0.0006**
Ratings of satisfaction based on VAS responses			
Written research project and submission to IRB	9 (3-10)	9 (0-10)	0.867
Writing the scientific manuscript	9 (0-10)	9 (0-10)	0.318
Submitting the manuscript to a scientific journal	8 (0-10)	8 (0-10)	0.857
Mentorship supervision	10 (5-10)	10 (2-10)	0.512
Your profession in the past year	8 (0-10)	9 (1-10)	**0.0113**
The graduate program	9 (1-10)	9 (2-10)	0.342
Workload (hours/week)			
≤20 hours	8 (7)	4 (4)	0.560
20-40 hours	48 (40)	25 (28)	0.079
>40 hours	64 (53)	62 (68)	**0.0340**
Patients/week			
≤50 patients	51 (42)	45 (50)	0.331
50-100 patients	38 (32)	31 (34)	0.768
100-200 patients	7 (6)	3 (3)	0.520
>200 patients	1 (1)	2 (2)	0.579
No patient supervision	23 (19)	10 (11)	0.127
Salary in the past year			
≥15 minimum wages/month	45 (37)	51 (56)	**0.0083**
Scientific meeting participation in the past year	105 (87)	86 (94)	0.100
Published thesis	99 (82)	67 (74)	0.129
Faculty service	40 (33)	42 (46)	0.065
Main issues related to clinical practice in the past year			
Long work hours	54 (45)	35 (38)	0.398
Poor social life	46 (38)	28 (31)	0.308
Harassment	7 (6)	2 (2)	0.305
Low salary	45 (37)	22 (24)	0.052

Results are presented as n (%) and median (range), VAS - visual analog scale (0=“no satisfaction at all” and 10=“complete overall satisfaction”), IRB – institutional review board.

**Table 4:** Demographic data, ratings of satisfaction, workload, patients/week, salary, scientific meeting participation, published research paper, faculty service, and main issues related to profession according to years of clinical practice in two groups of master’s-, doctoral-, or post-doctoral-level health professionals: women and men.

Variable	Women (n=164)	Men (n=47)	*p*
Demographic data			
Current age, years	46 (28-71)	50 (29-69)	0.259
Years since master’s/doctoral/post-doctoral completion	7,5 (1-45)	13 (1-24)	0.068
Currently married/partnered	114 (69)	39 (83)	0.094
Number of children	2 (0-4)	2 (0-4)	0.828
Ratings of satisfaction based on VAS responses			
Written research project and submission to IRB	9 (0-10)	9 (3-10)	0.203
Writing the scientific manuscript	9 (0-10)	9 (3-10)	0.565
Submitting the manuscript to a scientific journal	8 (0-10)	8 (0-10)	0.981
Mentorship supervision	10 (5-10)	10 (2-10)	**0.0324**
Your profession in the past year	8 (0-10)	9 (3-10)	**0.0015**
The graduate program	9 (1-10)	9 (2-10)	0.273
Workload (hours/week)			
≤20 hours	12 (7)	0 (0)	0.072
20-40 hours	65 (40)	8 (17)	**0.005**
>40 hours	87 (53)	39 (83)	**0.0002**
Patients/week			
≤50 patients	77 (47)	19 (41)	0.507
50-100 patients	53 (32)	16 (34)	0.861
100-200 patients	6 (4)	4 (8)	0.239
>200 patients	3 (2)	0 (0)	1.000
No patient supervision	25 (15)	8 (17)	0.820
Salary in the past year			
≥15 minimum wages/month	61 (37)	35 (74)	**0.0001**
Scientific meeting participation in the past year	145 (88)	42 (89)	1.000
Published thesis	127 (77)	39 (83)	0.545
Faculty service	60 (37)	22 (47)	0.236
Main issues related to clinical practice in the past year			
Long work hours	63 (38)	26 (55)	**0.045**
Poor social life	56 (34)	18 (38)	0.607
Harassment	7 (4)	2 (4)	1.000
Low salary	58 (35)	9 (19)	**0.0496**

Results are presented as n (%) and median (range), VAS – visual analog scale (0=“no satisfaction at all” and 10=“complete overall satisfaction”), IRB – institutional review board.
